# Agriculture: Green Farming Equipment

**Published:** 2005-09

**Authors:** Graeme Stemp

California’s San Joaquin Valley, known for its rich harvests of grapes, tomatoes, and oranges, is also prone to smog and ground-level ozone created when the equipment that works the land combines with the natural topography. Now farmers can do their part to clear the air by using “green” farm machinery that boasts greater efficiency and cleaner fuels.

In July 2004, California legislators set new air quality regulations for farmers, forcing them to significantly reduce their emissions of potential greenhouse gases and fine particles. Farmers—a tough lobbying group—were previously exempt from state air regulations. But the machinery, dust, pesticide use, and other facets of farming make this industry one of the worst polluters.

The American Lung Association’s *State of the Air 2005* report ranked three California farm counties (Kern, Fresno, and Tulare) among the five worst in the nation for ozone and particle pollution. Such poor air quality has a major health impact, especially for young children. A study by the Central California Children’s Institute found that 15.7% of San Joaquin Valley children had asthma. Statewide, Fresno and Kings counties had the worst asthma rates, with more than 20% of children diagnosed.

The “Optimizer,” developed by Kevin McDonald, founder and president of Tillage International, offers one way to make farm-work more efficient. This multipurpose tiller comes in two models and does all the necessary tilling, planting, and herbicide application in one step. Farmers who once had to do multiple passes can work their fields in one or two passes, cutting down on the amount of tractor fuel needed.

McDonald says growers currently using the Optimizer estimate that the tiller, at $149,000–$189,000 depending on model, could pay for itself in under a year. Researchers at the University of California, Davis, tested the machinery and found that it saved 50% on fuel and 72% on time. Furthermore, the Optimizer is eligible for a Natural Resources Conservation Service grant through the U.S. Department of Agriculture, which could significantly cut the one-time purchase cost.

Another innovative way to cut emissions is to replace petroleum fuel with biodiesel. Biodiesel is made by refining vegetable oils such as those found in soybeans and rape-seed, and can be mixed with regular diesel in varying concentrations. In October 2002 the U.S. Environmental Protection Agency analyzed the emissions of a 20% biodiesel/ 80% petroleum diesel blend and found reduced emissions of particulate matter (–12%), unburned hydrocarbons (–20%), and carbon monoxide (–12%).

While a diesel engine will run using 20% or even 100% biodiesel, equipment manufacturers like New Holland and John Deere recommend only 2–5% biodiesel. But even a small amount counts when you’re as big as John Deere; the company announced in February 2005 it would begin using a 2% biodiesel blend as the preferred factory fill for all its diesel machinery.

If air concerns don’t convince farmers to invest in new products, simple economics may. A 1989 report by the California Air Resources Board noted that grapes, cotton, oranges, lemons, and beans grown in 1985 levels of air pollution lost 16–29% in yield and size as a direct result of smog.

## Figures and Tables

**Figure:**
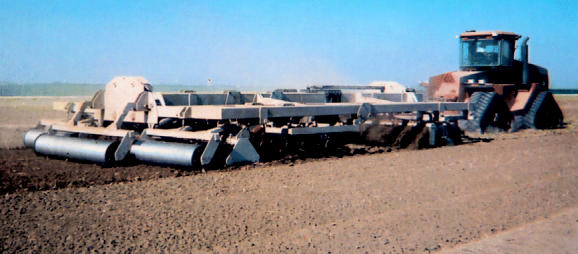
Easier on the air. The Optimizer is part of a new generation of green farm equipment.

